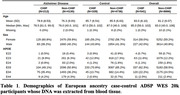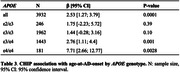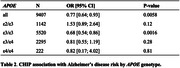# Clonal hematopoiesis is associated with a seven‐year delay in the onset of Alzheimer’s disease in *APOE* ε4/ε4 cases

**DOI:** 10.1002/alz.095398

**Published:** 2025-01-09

**Authors:** Yann Le Guen, Junyoung Park, Lia Talozzi, Michael E. Belloy, Michael D Greicius

**Affiliations:** ^1^ Stanford University, School of Medicine, Stanford, CA USA; ^2^ Washington University in Saint Louis, Saint Louis, MO USA

## Abstract

**Background:**

Recently, clonal hematopoiesis of indeterminate potential (CHIP) was found to be associated with a reduced risk of Alzheimer’s disease (AD) and somatic mutations found in the blood of CHIP carriers were also in microglia‐enriched brain samples (Bouzid et al., 2023, *Nature Medicine*). We aimed to validate this finding in a larger dataset, explore the effect across *APOE* genotypes, and examine the association of CHIP with age‐at‐onset.

**Method:**

Bouzid et al. (2023) analyzed the Alzheimer’s Disease Sequencing Project (ADSP) whole‐exome sequencing (WES) 10k subjects release and limited their analysis of this cohort to ε3/ε3 individuals, while we analyzed the new ADSP WES 20k subjects release and considered all *APOE* genotypes. We identified CHIP variants from joint‐called vcf files focusing on genes and variants provided in the previous study appendix. We further filtered variants requiring a median read depth above 30 among carriers and variant allele fraction below 40%. As in Bouzid et al., we restricted our analysis to European ancestry participants whose DNA was extracted from blood (**Table 1**). Using the generalized linear model implemented in the statsmodel Python package, we tested the association with diagnosis (Binomial family) and age‐at‐onset (Gaussian family) across and by *APOE* genotype. Analyses were adjusted for sex, and the first 3 principal components accounting for genetic ancestry. Additionally, the analyses considering all *APOE* genotypes were adjusted for ε2 and ε4 dosages.

**Result:**

We confirmed CHIP association with reduced AD risk in ε3/ε3 individuals (OR = 0.68 [0.54; 0.86], p = 0.0016), and observed a similar direction of effect across *APOE* genotypes (OR = 0.77 [0.64; 0.93]; p = 0.0058), except in ε2/ε3 (**Table 2**). In addition, we found that CHIP was associated with 2.5 years delayed age‐at‐onset across *APOE* genotypes (b = 2.53 [1.27; 3.79]; p = 0.0001, **Table 3**), with a marked 7.7 years delayed onset in ε4/ε4 cases (b = 7.71 [2.66; 12.77]; p = 0.0028).

**Conclusion:**

Taken together, these results provide stronger human genetic evidence that CHIP is associated with reduced AD risk and delayed age‐at‐onset, particularly in *APOE*‐ε4 carriers. These findings corroborate the notion that rescuing microglial phagocytic capacity during aging may be a therapeutic avenue for AD, most notably in *APOE*‐ε4 carriers.